# Experiences of and resistance to multiple discrimination in health care settings among transmasculine people of color

**DOI:** 10.1186/s12913-022-07729-5

**Published:** 2022-03-21

**Authors:** Madina Agénor, Sophia R. Geffen, Dougie Zubizarreta, Raquel Jones, Shane Giraldo, Allison McGuirk, Mateo Caballero, Allegra R. Gordon

**Affiliations:** 1 Department of Behavioral and Social Sciences, Brown University School of Public Health, Providence, RI USA; 2 Center for Health Promotion and Health Equity, Brown University School of Public Health, Providence, RI USA; 3 The Fenway Institute, Fenway Health, Boston, MA USA; 4 Johns Hopkins University School of Nursing, Baltimore, MD USA; 5 Center for Health Equity Education and Advocacy, Cambridge Health Alliance, Cambridge, MA USA; 6 Department of Epidemiology, Harvard T.H. Chan School of Public Health, Boston, MA USA; 7 Department of Psychology, University of Massachusetts Boston, Boston, MA USA; 8 Department of Sociology, Simmons University, Boston, MA USA; 9 Youth Community Advisory Board, Tufts University, Medford, MA USA; 10 Department of Counseling Psychology, University of Wisconsin Madison, Madison, WI USA; 11 Department of Communication Studies, Northeastern University, Boston, MA USA; 12 Department of Community Health Sciences, Boston University School of Public Health, Boston, MA USA; 13 Division of Adolescent/Young Adult Medicine, Boston Children’s Hospital, Boston, MA USA; 14 Department of Pediatrics, Harvard Medical School, Boston, MA USA

**Keywords:** Transgender health, People of color, Health care, Structural barriers, Discrimination, Cissexism, Racism, Weight-based discrimination, Ableism, Resistance

## Abstract

**Background:**

Research shows that transmasculine people experience discrimination based on their gender identity and/or expression (i.e., cissexism) while obtaining health care. However, studies examining the experience of other forms of discrimination in health care settings among diverse subgroups of transmasculine individuals, including those from minoritized racial/ethnic backgrounds, are very limited.

**Methods:**

Guided by intersectionality, we designed a qualitative research study to explore how transmasculine people of color experience—and resist—multiple, intersecting forms of discrimination in health care settings. Guided by a purposive sampling strategy, we selected 19 transmasculine young adults of color aged 18–25 years to participate in 5 mini-focus groups conducted between February and May 2019 in Boston, MA. Focus group transcripts were analyzed using a template style approach to thematic analysis that involved both deductive and inductive coding using a codebook. Coded text fragments pertaining to participants’ experiences of health care discrimination were clustered into themes and sub-themes.

**Results:**

Transmasculine people of color described experiencing notable challenges accessing physical and mental health care as a result of structural barriers to identifying health care providers with expertise in transgender health, finding providers who share one or more of their social positions and lived experiences, and accessing financial resources to cover high health care costs. Further, participants discussed anticipating and experiencing multiple forms of interpersonal discrimination—both independently and simultaneously—in health care settings, including cissexism, racism, weight-based discrimination, and ableism. Moreover, participants described the negative impact of anticipating and experiencing multiple interpersonal health care discrimination on their health care utilization, quality of care, and mental and physical health. Lastly, participants discussed using various strategies to resist the multiple, intersecting forms of discrimination they encounter in health care settings, including setting boundaries with health care providers, seeking care from competent providers with shared social positions, engaging in self-advocacy, drawing on peer support during health care visits, and obtaining health information through their social networks.

**Discussion:**

Efforts are needed to address cissexism, racism, weight-based discrimination, ableism, and other intersecting forms of discrimination in clinical encounters, health care institutions and systems, and society in general to advance the health of transmasculine people of color and other multiply marginalized groups.

**Supplementary Information:**

The online version contains supplementary material available at 10.1186/s12913-022-07729-5.

## Introduction

Studies show that a substantial proportion of transgender men (i.e., men assigned female at birth [AFAB]) and women (i.e., women assigned male at birth [AMAB]) and gender diverse (e.g., non-binary, genderqueer, gender fluid) individuals—including transmasculine people (i.e., transgender men and gender diverse AFAB people with masculine gender identities)—experience bias, prejudice, and discrimination based on their gender identity (i.e., cissexism) while obtaining health care [1–6]. For example, in the 2015 U.S. Transgender Survey (USTS), a large national survey of transgender and gender diverse individuals, one-third of participants who reported seeing a health care provider in the past year had at least one negative experience related to their gender identity and/or expression—including denial of services; verbal, physical, and sexual abuse; intrusive, non-respectful care; and having to educate their health care provider about transgender health [2]. Experiences of cissexism in health care settings have notable negative effects on health care access and utilization among transgender and gender diverse individuals [1, 2, 7, 8]. Indeed, research shows that large numbers of transgender and gender diverse individuals avoid and postpone needed health care, including when sick or injured, as a result of past experiences and fears of cisexism [1, 2, 7, 8]—which in turn contributes to poor health outcomes in these marginalized and underserved populations [1, 2, 9].

Intersectionality [10–14], an analytical framework rooted in Black feminist theory and practice [15–18], suggests that multiply marginalized transgender and gender diverse people, such as Black, Native, Latinx/e, Asian, and other transgender and gender diverse people of color, experience—and resist—not only cissexism but also other intersecting forms of discrimination, such as racism. Specifically, intersectionality postulates that multiply marginalized individuals’ lived experiences are shaped by experiences of and resistance to mutually constitutive, unequal power relations (e.g., racism, (cis)sexism, classism, heterosexism)—which are rooted in reciprocal systems of exploitation and oppression (e.g., white supremacy, patriarchy, capitalism, colonialism) and result in experiences of discrimination that are both unique and more than the sum of their parts [10–14]. However, research on discrimination and health care among transgender and gender diverse people has largely focused on cissexism, to the exclusion of other reciprocally constructing forms of discrimination, among samples of predominately white individuals [1–6].

Nonetheless, a small body of research shows that transgender and gender diverse people of color have unique and compounding experiences of multiple forms of discrimination in health care settings [1, 2, 19]. First, studies indicate that transgender and gender diverse people of color experience higher levels of cissexism in health care settings, including emergency rooms, doctors’ offices, hospitals, and ambulances, compared to their white counterparts [2, 3]. For example, using data from the 2010 National Transgender Discrimination Survey, Kattari et al. found that 26.1% of transgender and gender diverse people of color experienced cissexism in a doctor’s office or hospital, relative to 18.5% of white participants [3]. Moreover, 2015 USTS data show that, while 34% of white participants reported at least one negative experience with a health care provider in the past year as a result of their gender identity and/or expression, 50% and 40% of Native and Middle Eastern participants, respectively, reported such an experience [2]. Second, research indicates that the health care experiences of transgender and gender diverse people of color are simultaneously impacted by not only cissexism but also racism [3, 19–21]. For example, in a qualitative study of transgender people of color in Chicago, IL, Howard et al. found that participants reported negative health care experiences as a result of both cissexist and racist stereotypes among health care providers and difficulty identifying providers who met their needs and preferences related to both gender identity and race/ethnicity [19]. Compounding experiences of cissexism and racism in health care settings may in turn contribute to the higher levels of avoidance of care due to fear of mistreatment among transgender and gender diverse people of color relative to their white counterparts [2].

Research indicates that access to and utilization of health care is undermined by not only cissexism and racism but also classism, ableism, xenophobia, and weight-based discrimination, among others [2, 22–26]. However, studies examining the impact of these forms of discrimination on the health care experiences of multiply marginalized transgender and gender diverse people is extremely limited. Indeed, we could not identify any published study on discrimination and health care among transgender and gender diverse individuals explicitly focusing on classism, ableism, or xenophobia. Further, to our knowledge, only one published study has specifically investigated the negative effects of weight-based discrimination on the health care experiences of lesbian, gay, bisexual, transgender, and queer (LGBTQ) people in general [27]. Thus, we designed a qualitative research study to contribute to the literature on intersectionality, discrimination, and access to and use of health care among multiply marginalized transgender and gender diverse individuals—focusing on how transmasculine people of color, a particularly understudied and underserved subgroup, experience and resist cissexism, racism, and other forms of discrimination in health care settings.

## Methods

### Participant recruitment

Using a purposive sampling strategy [28–30], we recruited individuals who met the following eligibility criteria: identified as a person of color, including but not limited to Black, Latinx/e, Native, and/or Asian; identified as transmasculine, including but not limited to a transgender man or a masculine gender diverse AFAB person; were between ages of 18 and 25 years; and resided in the greater Boston area. Using maximum variation sampling [28–30], we recruited participants from diverse gender identity and racial/ethnic backgrounds in order to capture the perspectives and experiences of a broad range of transmasculine people of color.

We recruited participants between October 2018 and May 2019 by sharing study flyers with community-based organizations, community health centers, college student groups, email listservs, and Facebook groups that serve transmasculine young adults of color in the greater Boston area. Additionally, participants were asked to disseminate the study flyer to individuals in their social networks using a chain referral sampling strategy. Further, we shared information about our study with our personal and professional networks, who then shared this information with transmasculine individuals who may be eligible for and interested in participating in the study [28–32]. Lastly, drawing on the principles of community-based participatory research, we convened and collaborated with a Youth Community Advisory Board (YCAB) composed of three transmasculine young adults of color who helped us identify potential recruitment sites and partners and disseminated the study flyer within their social networks [31, 33, 34].

### Data collection

We conducted mini focus group discussions (*N* = 5) with 3 to 5 transmasculine young adults of color each (*n* = 19) to elicit detailed and nuanced information on their sexual and reproductive health care experiences and their multilevel social determinants [35]. We chose to conduct mini focus groups to allow members of this multiply marginalized population—whose needs, concerns, and experiences are often ignored—ample time to share their perspectives on a variety of sensitive topics, which are often more easily discussed in smaller groups that facilitate rapport and comfort [35]. We determined our sample size a priori based on research indicating that thematic or meaning saturation is generally reached with 2–6 focus groups [36–38]. Indeed, after 5 mini-focus groups, we found no new theme pertaining to our research question and were able to generate a rich, comprehensive understanding of each of our themes and subthemes [36]. All focus group participants provided written informed consent at the start of each group.

Focus groups were conducted between February and May 2019 in Boston, MA using a semi-structured focus group discussion guide (Appendix) [35]. The discussion guide included open-ended questions and probes pertaining to the following topics: sources of sexual and reproductive health information, sexual and reproductive health beliefs and risk perceptions, and sexual and reproductive health care attitudes, needs, preferences, and experiences. Focus group discussion guide questions included: “Tell me about a time when your transmasculine status, race/ethnicity, or other aspects of your identity affected your health care,” “Tell me about your experience discussing HIV and STIs with health care providers since identifying as transmasculine,” and “Tell me about your experience getting tested for HIV and STIs since identifying as transmasculine.” The guide was informed by the scientific literature on sexual and reproductive health among transmasculine individuals and reviewed by members of the YCAB, who provided oral and written input and feedback from their perspectives. The guide was also reviewed by members of a lesbian, gay, bisexual, transgender, and queer (LGBTQ) health research working group, which includes experts in transgender health, adolescent and young adult health, and sexual and reproductive health.

Each focus group was conducted in person by an academic member of the research team with expertise in qualitative public health research or a trained member of the YCAB, both of whom were LGBTQ (i.e., queer cisgender woman, transmasculine individual) people of color (i.e., Latinx/e, Black). A trained member of the research team also took detailed notes during the focus groups. All focus groups were conducted at a transgender-inclusive social justice organization, LGBTQ youth community-based organization, or LGBTQ community health center, which helped facilitate participant comfort and promote group rapport and discussion. Focus groups were conducted in English and audio-recorded. Focus groups lasted between 93 and 111 min (mean: 99 min). At the end of each focus group, participants completed a brief demographic survey to help contextualize qualitative research findings. Participants received a $45 gift card for their time. All research activities were reviewed and approved by the Tufts University Social, Behavioral, and Educational Research Institutional Review Board. All methods were carried out in accordance with relevant guidelines and regulations.

### Data analysis

Audio-recordings of the focus groups were transcribed verbatim by a professional transcription company and entered into Dedoose (version 8.3.19, Manhattan Beach, CA) for analysis. Focus group transcripts were analyzed using a template style approach to thematic analysis that involved both deductive and inductive coding using a codebook [39–41]. Data analysis began with immersion in the data, followed by codebook development, testing, and refinement. The codebook was developed collaboratively among research team and YCAB members and included both deductive codes based on the scientific literature and inductive codes based on the focus group transcripts. Two independent coders each applied the codebook to two focus group transcripts to test its fit to the data. Codes were then merged, refined, and discarded, and the revised codebook was applied independently by each coder to all five transcripts. Coding discrepancies were discussed and resolved by consensus, and the codebook was further refined and reapplied to the data based on these ongoing research team discussions [39–43].

For the present manuscript, coded text fragments pertaining to participants’ experiences of health care discrimination were clustered into themes and sub-themes [39, 42, 44–46]. Relevant quotes were organized according to the theme(s) and sub-theme(s) to which they pertained using a data analysis matrix, which allowed for further refinement and comparison of themes and sub-themes across and within focus groups and participants by gender identity and race/ethnicity [47]. Memo writing and regular research team meetings were also used to facilitate the identification and refinement of themes and sub-themes [48]. Focus group transcripts were then reviewed to ensure that all relevant coded excerpts were included in the analysis, study findings accurately represented the data, and all relevant themes had been identified [49]. All research team and YCAB members provided feedback on the themes and sub-themes, which were also reviewed by members of a LGBTQ health research working group composed of experts in transgender health, adolescent and young adult health, and sexual and reproductive health.

### Reflexivity

Throughout the research process, members of the research team engaged in reflexivity, which was facilitated by writing memos and engaging in team discussions. Memos and discussions focused on examining the effects of research team members’ professional background, social positions, and values as well as the impact of social, economic, and political contexts on both data collection and analysis. In particular, data collection and analysis were conducted by academic researchers with training in qualitative public health research and in the structural and social determinants of health inequities. Research team members had diverse sexual orientation (e.g., queer, bisexual), gender (e.g., cisgender woman, transmasculine individual), and/or racial/ethnic (e.g., Black, Latinx/e, white) identities and had strong commitments to racial, social, and economic justice. The research team engaged with study participants and YCAB members as co-creators of knowledge and meaning with expertise in the research question of interest. To that end, YCAB members provided input and feedback on all data collection procedures and on the themes and sub-themes resulting from data analysis and were included as co-authors of the present manuscript. In particular, one trained YCAB member took on a more in-depth role in data collection and analysis by serving as a focus group discussion moderator and coding transcripts. Additionally, in light of the social, economic, and political marginalization and oppression experienced by transmasculine people of color in U.S. society in general and the health care system in particular, all focus groups were conducted in partnership with community-based organizations and community health centers that serve individuals with minoritized gender and racial/ethnic backgrounds.

## Results

### Participant sociodemographic characteristics

Table 1 shows that study participants ranged in age from 18–25 years, with a mean age of 22 years. Most participants identified as non-binary (37%; *n* = 7) in terms of gender identity, queer (42%; *n* = 8) in terms of sexual orientation identity, and Black (58%; *n* = 11) in terms of race/ethnicity. Further, most participants were working full-time (47%; *n* = 9), and the majority had some college education or more (84%; *n* = 16) and were enrolled in a private health insurance plan (63%; *n* = 12). Additionally, the majority of participants received care at a community health center (63%; *n* = 12) and from a Nurse Practitioner or Registered Nurse (74%; *n* = 14).

**Table 1:** Sociodemographic characteristics of transmasculine people of color (*n* = 19) from five focus groups

Variable	n	%
Age (mean, years): 22		
Gender identity		
Man or transgender man	4	21
Transmasculine	3	16
Gender non-conforming	1	5
Non-Binary	7	37
Gender fluid	1	5
Agender	2	11
Another gender identity	1	5
Sexual orientation identity^*^		
Queer	8	42
Lesbian	4	21
Bisexual	2	10
Gay	3	16
Pansexual	8	42
Asexual	2	11
Not sure	1	5
Race/ethnicity^*^		
Black	11	58
Latinx	5	26
Multiracial	5	26
Asian	4	21
White	4	21
Native	1	5
Arab or Middle Eastern	1	5
Another race/ethnicity	1	5
Employment status^*^		
Working for pay, full-time	9	47
Working for pay, part-time	5	26
Not working for pay	1	5
Student	6	32
Educational attainment		
High school diploma	2	11
High school diploma or GED	1	5
Some college education/Associate’s degree	9	47
Bachelor’s degree or more	7	37
Health insurance		
Private health insurance	12	63
MassHealth (i.e., Medicaid)	5	26
Uninsured	2	11
Usual source of care^*^		
Private doctor’s office	6	32
Community health center	12	63
Hospital clinic	1	5
Planned Parenthood clinic	2	11
None	1	5
Another source of care	2	11
Usual health care provider seen^*^		
Physician (MD)	7	37
Nurse (RN or NP)	14	74
Physician Assistant (PA)	2	11
None	1	5

^*^ Response categories are not mutually exclusive

*Note*. Percentages may not add to 100% due to rounding error and non-mutually exclusive response categories

### Research findings

**Fig. 1:**
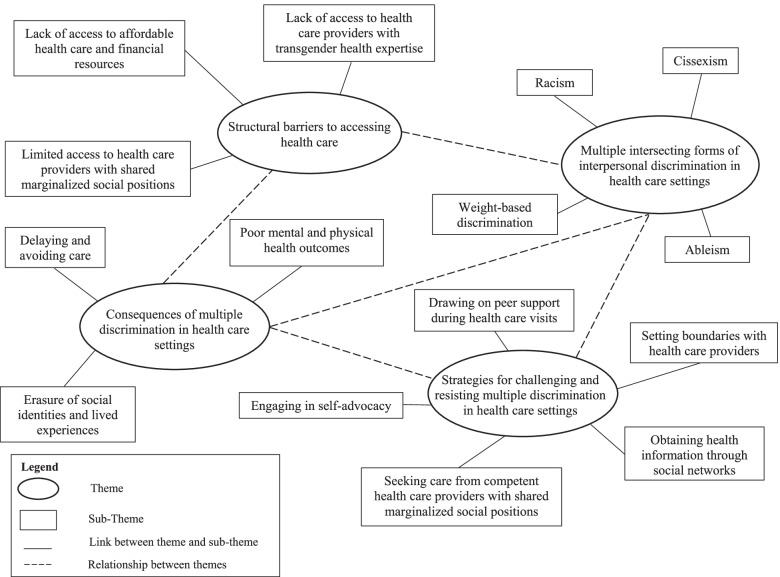
Thematic map of themes and sub-themes pertaining to the experiences of and resistance to multiple discrimination in health care settings among transmasculine people of color (*n* = 19) from five focus groups

We identified four major themes across the focus group transcripts: 1) structural barriers to accessing health care as transmasculine people of color; 2) anticipating and experiencing multiple intersecting forms of interpersonal discrimination in health care settings; 3) consequences of multiple discrimination in health care settings; and 4) strategies for challenging and resisting multiple discrimination in health care settings (Table 2, Fig. 1).

**Table 2:** Titles and definitions of themes pertaining to the experiences of and resistance to multiple discrimination in health care settings among transmasculine people of color (*n* = 19) from five focus groups

Theme	Title	Definition
1	Structural barriers to accessing health care as transmasculine people of color	Notable challenges accessing physical and mental health care as a result of lack of access to health care providers with expertise in transgender health, limited access to health care providers with one more shared marginalized social positions and related lived experiences, and lack of access to affordable health care and financial resources
2	Anticipating and experiencing multiple intersecting forms of interpersonal discrimination in health care settings	Anticipating and experiencing multiple intersecting forms of interpersonal discrimination—both independently and simultaneously—while navigating various health care settings, including cissexism, racism, weight-based discrimination, and ableism
3	Consequences of multiple discrimination in health care settings	Negative impact of anticipating and experiencing multiple intersecting forms of interpersonal discrimination in health care settings on participants’ health care utilization, quality of care, and mental and physical health
4	Strategies for challenging and resisting multiple discrimination in health care settings	Use of various strategies to challenge and resist multiple intersecting forms of discrimination in health care settings, including setting boundaries with health care providers, seeking care from competent health care providers with shared marginalized social positions, engaging in self-advocacy, drawing on peer support during health care visits, and obtaining health information through social networks

#### Theme 1. Structural barriers to accessing health care as transmasculine people of color

Most study participants described experiencing notable challenges accessing physical and mental health care as a result of structural barriers to identifying health care providers with expertise in transgender health, finding health care providers who share one or more of their social positions and lived experiences, and accessing financial resources to cover high health care costs.

##### Lack of access to health care providers with expertise in transgender health

The majority of participants discussed the challenges they faced identifying health care providers with expertise in transgender health, which in turn undermined their access to and use of both mental and physical health care. For example, an Arab and multiracial transmasculine participant noted: “[I] disclosed [my gender identity] to my therapist and then 2 months later he said he didn't feel qualified to take care of someone with a non-binary gender because he felt like there was something exotic about it. He was very clear that he felt like he was going to misstep and then hurt me, and it would really stress him out. I felt like I was being released into the ocean, no support at all. And it was literally because of my identity.” Further, explaining his avoidance of sexual and reproductive health care, a Latinx/e transgender man explained: “I’ve never been to the OB/GYN. And the reason I haven’t gone at all is because I’m very afraid of it being awkward and having to explain things or what T [testosterone] does. And I’m not looking forward to that, because it’s very frustrating, because it makes me feel like I don’t exist. It leaves me feeling really pessimistic. There’s so few of us, why ever would doctors be trained? They just don’t think it’s important.”

##### Limited access to health care providers with one or more shared marginalized social positions

Most transmasculine people of color in our study, including those who reported finding health care providers with expertise in transgender health, reported difficulty identifying health care providers who shared one or more of their marginalized social positions and related lived experiences. Many participants reported experiencing barriers to identifying and receiving care from health care providers of color, for whom they expressed a preference. For example, a Black non-binary participant stated: “I would prefer to see people of color. It’s nice when I don’t feel a cultural barrier between myself and the provider. Or just seeing someone who looks like me, so that I know they’re not denying me care because of the color of my skin or anything like that.” Additionally, several participants also described the challenges they faced identifying and receiving care from providers who shared more than one of their social positions as transmasculine people of color, for which they expressed a preference. For example, a Black, Native, and multiracial agender participant noted: “I’m trans, I’m poly[amorous], I’m into kink. I am Brown/Black and AFAB [assigned female at birth] and transmasculine even though I identify as agender. I don’t want a therapist that’s white. And so, that’s a very narrow pool. Finding niche doctors is really hard. And then all the actual ones that do fit that are already taken up because they’re already so limited.”

A few participants discussed the conflicts they experienced among their different social positions and compromises they had to make in selecting a health care provider who met their needs and preferences as transmasculine people of color. For example, an Asian transmasculine participant noted: “[My therapist] was trying to figure out who to transition me to when she left, and she was like, ‘Well, there’s this person who’ll understand your culture. She has a South Asian heritage,’ or, ‘There’s this person who understands the queer side.’ You’ve got to pick.” Similarly, a Black agender participant mentioned: “It’s scary to try to find these doctors. Sometimes I have to compromise one box they don’t tick off, as long as they tick off the rest. I have to think about, ‘What’s more important for me right now?’ [rather] than think about ticking off all the boxes, because no one’s going to check off all the boxes, usually. For example, I have a therapist that’s non-binary and trauma-informed, but they’re white. I want a therapist of color, but that’s okay, because they ticked off at least two of the boxes.”

##### Lack of access to affordable health care and financial resources

Some participants reported facing pronounced structural economic barriers to accessing health care due to the high cost of health care and a lack of access to financial resources. For example, a Black Caribbean transgender man explained: “I think the main thing is how serious the situation is, and if I can afford it. So, if I feel like I really need to be seen for something that’s kind of worrying me to the point where I’m stressing out, then I’ll find a way and try to pay it and make an appointment. But if I think, ‘I can deal with this,’ then I’m usually trying to save my money.” Further, an Arab and multiracial transmasculine participant noted: “Seeing a doctor is kind of an ordeal financially. And also, just feeling like navigating the whole thing [health care system] and not knowing how much it costs ever.” Similarly, a Black, white, and multiracial demi-girl stated: “I’m hesitant to go, but I’ll say ‘Well, if I need to.’ But the bigger problem is just finances. And also the fact that I’m unemployed. It’s not always directly, ‘Oh I won't go to the doctor because they’re going to discriminate against me there.’ It’s like I can't afford to pay because I don’t have a job because anywhere I want to work doesn’t want to hire someone like me.”

#### Theme 2. Anticipating and experiencing multiple intersecting forms of interpersonal discrimination in health care settings

Participants discussed anticipating and experiencing multiple, intersecting forms of interpersonal discrimination as transmasculine people of color navigating various health care settings.

##### Cissexism

Most participants reported anticipating cissexism in the context of health care. For example, a Black non-binary participant noted: “It’s also nerve-racking because you never know how a clinician is going to treat you and if they’re going to change after you come out to them, like, ‘Oh, is this person secretly anti-LGBT? And now I told them, and now they’re not a safe health care provider for me.’ Or are they going to think I’m crazy and not listen to me? Or invalidate my experiences?” Similarly, a Black, white, and multiracial demi-girl explained: “The worst part is just not knowing if they’re going to regard me the right way. Like acknowledge me how I want to be acknowledged. Whether it’s my pronouns and my name or just the way they’re looking at me or are asking questions. That’s always something I have to worry about and anticipate or prepare myself for. It's usually not to the extent of feeling mocked or ridiculed but sometimes it is, and I just kind of have to prepare myself for like, ‘Oh these people are judging me when I walk in the door.’”.

Further, the majority of participants reported either personally experiencing or hearing about peers experiencing cissexism—including intrusive, disrespectful, and inappropriate care as a result of their transgender or gender diverse status—during clinical encounters. For example, a Black, Latinx/e, and white non-binary participant mentioned: “I have scoliosis and fibromyalgia so I will have to go see a back doctor, and then they’re just like, ‘Oh so I see you’re like gender…’ and I was like, ‘I don’t want to talk about that! I want to talk about my back.’ And then they keep asking me questions about my gender when it has nothing to do with my back.” Moreover, referring to their peers’ experiences of cissexism in sexual and reproductive health care settings, a Latinx/e and Native transmasculine participant noted: “I’m really nervous to get a Pap smear. [I’ve] just heard some horror stories from friends who have doctors who are very confused by the genitals of people who went on T [testosterone]. Also, one of my friends got referred to a psych person because they thought they were a man who thought he needed a Pap smear. They thought they were a cis[gender] dude.”

In contrast, several participants described feeling affirmed and validated when receiving care from health care providers with knowledge of and experience in transgender health who used gender-inclusive language. For example, a Latinx/e and Native transmasculine participant explained: “My PCP [primary care provider] was really good. She used this awesome language where she was like, ‘Have you been with any sperm-producing people?’ And I loved that, because I used to identify as a lesbian, and I only had sex with people who identified as women. And since then, that’s changed a lot and the bodies of the people that I sleep with are much more diverse now. And so that was really helpful language for me personally and felt validating not only of me but of my partners.” Similarly, an Asian non-binary participant mentioned: “She doesn’t ever gender any body parts or myself. And I was kind of nervous going to her as a non-binary person who wanted to start T [testosterone], because I wasn’t sure if I would have to pretend that I was a trans man or something like that. She was very understanding and said she has a lot of other patients like that, so I just think she has a lot of experience.”

##### Racism

Many participants in our study—notably, all of the Black participants—reported experiencing racism in health care settings, including erroneous racist health care provider beliefs about their sexual behavior and level of pain tolerance, which they described as being further compounded by (cis)sexism. For example, a Black, white, and multiracial demi-girl explained: “When I had a different psychiatrist before, he would always make these comments regarding me as aggressive. He’d hypersexualize me and just make me more mature in his head than I actually was because of my racial identity.” Moreover, a Black agender participant observed: “There’s this common misconception that Black folks, especially Black people who are assigned female at birth, are less likely to feel pain. [That] is a consistent thing that I do notice [when seeking care].” Further, a Black gender non-conforming participant stated: “I specifically only ask other people of color about health care. Because I feel like the people who generally tend to have negative experiences, specifically at [LGBTQ health clinic], are always people of color, especially the Black people. If doctors are, you know, hypersexualizing you because you’re Black, white people won’t notice that.” This participant continued: “But I think that Black, especially Black transgender people, should be able to go into a health space and be able to ask for sexual health services and testing without the ‘Oh, so you been [having sex.]’ That always seems to happen.”

Moreover, several participants mentioned that they experienced less or no racism relative to their peers with darker skin tones as a result of their lighter skin color and their proximity to whiteness. For example, a Black gender non-conforming participant observed: “I know that I’m lucky in that, as a light-skinned Black person, I don’t deal as much with hyper-sexualization in health spaces.” Similarly, referring to their darker-skinned partner, a Black, white, and multiracial demi-girl observed: “And so sometimes, I notice that at similar places that we may go for certain care, they would acknowledge us differently because they feel more favoritism towards me because of my lighter skin and whatever else.” Moreover, referring to being perceived as white despite not identifying as such, a Latinx/e transgender man mentioned: “Because I’m white-passing, I haven’t had experiences where doctors are outwardly rude or anything.” Further, with regard to their experiences of racism, which were shaped by not only their skin color but also their parent’s race/ethnicity, both their own and their parent’s social class, and their gender expression, a Black, white, and multiracial non-binary individual explained: “I feel like I’ve had a very different experience. I have parents that are both white. And my mom would always […] come with me to the doctor when I was growing up. So, I definitely had that, ‘this is the child of this middle-class, white woman. And so, I think that was a good, pretty strong barrier [against racism].” Referring to their current experiences of racism and other discrimination in health care settings, they continued: “I think it’s easy for me to go in and be like, ‘Yeah, I’m a well-educated, maybe light skinned, maybe Italian, woman.’ I can have people read me that way. And then know that people […] are going to listen to me and respect me, and do whatever I want them to do.”

##### Weight-based discrimination

Several, mostly Black participants also reported experiencing weight-based discrimination in health care settings, including health care providers dismissing their health concerns and needs. For example, a Black non-binary participant described interacting with health care providers who attributed their health concerns to their weight and prioritized addressing their weight over their health concerns. They noted: “A lot of the time, [it’s] like, ‘Oh, I’m in pain.’ And they go like, ‘Did you try losing weight first?’ They continued: “Or if I have cramps, ‘You should lose weight, it will get better.’ I'm like, ‘What if you just gave me a solution that would work? What if I didn’t want to lose weight? What if I’m fine the way I am?’” Similarly, a Black gender non-conforming participant explained: “I have chronic joint pain literally all the time. I get random dizzy spells. I have all these symptoms that I don’t know what the cause of them are. And I’ve tried bringing it up with my PCP [primary care provider], and she would send me to 10 different specialists. All of them would be like, ‘Hm. Looks good to me. You’re fine.’ I’m like, ‘No, it’s not. No.’ So finding a doctor that would actually take my symptoms seriously and wouldn’t, just chalk it up to me being fat… I still haven’t really found any competent doctors who take chronic illness seriously in general, never mind for folks who are fat.”

Moreover, a few participants described how anticipating and experiencing weight-based discrimination in health care settings was a deterrent to seeking care and a barrier to obtaining high-quality care. For example, a Black agender participant explained how weight-based stigma and discrimination were a deterrent to seeking care and could also promote poor health: “But the fatphobia definitely has been very…I have been scared to see doctors before, at a certain weight. I have lost a lot of weight. And because of being on T [testosterone] and working with children, I’ve lost 70 pounds. And it’s kind of good, because yeah, I lost 70 pounds through mostly healthy means. But sometimes, I lose weight rapidly, and it’s because I’m too depressed to eat. And it’s hard to find doctors that can understand that two things can exist. That weight loss can exist because you’re working out a lot and you’re eating healthier, but it can also exist because you’re sick.”

Lastly, some participants described how health care providers use their weight as an excuse to deny them gender-affirming surgery. For example, a Black and Latinx/e non-binary participant noted: “The best doctor to go to is [doctor’s name], but he doesn’t take you if you’re over a certain BMI. And if you… even if you express to him, you know, ‘Actually, we probably shouldn’t have conversations about my weight. I am recovering from an eating disorder. We really can’t talk about this.’ He’ll still tell you, ‘Well, you need to lose weight if you want me to do the surgery for you.’” In contrast, a Black, Native, and multiracial agender participant described receiving care that was affirming of both their gender and weight and respectful of their bodily goals. They explained: “I went swimming for the first time in February shirtless. It was great, it was really nice. And it’s like they’re the right size. I’m a fat person, I shouldn’t have a flat chest. I should have something because that’s how fat works on bodies. I’m really glad that the doctors and the people I interacted with were knowledgeable enough for what I needed to help me achieve what I wanted.”

##### Ableism

A few participants reported experiencing disability-related discrimination in health care settings. For example, a Black, white, and multiracial demi-girl mentioned: “I am autistic and so something that I do to regulate myself is I do tactile stims. And so it’s been a big thing where I’ve gotten medical abuse where they’ve tried to limit that and not allow me to do stuff like that, which would lead to panic attacks and stuff for me.” Additionally, a Black and Latinx/e genderfluid participant noted: “I’m young and [health care providers] don’t really think young people have chronic pain. So every time when they ask me [about] my pain, and I tell them about my issue with my feet or anything else, they’re kind of just like, ‘Well, I don’t know what to do’ or they send me to [another medical center].” Further, referring to the compounding negative effects of ableism, weight-based discrimination, (cis)sexism, and anti-Black racism in particular on the health care experiences, a Black gender non-conforming participant explained: “I feel like every part of my identity just kind of stacks on top and having this cute little brick wall that means that I won’t get the care that I need when I go to the doctor. Being fat usually means that my doctors are going to either attempt to chalk up whatever is wrong with me [to me] being fat.” They continued: “I’m technically diagnosed with ADHD, but that’s the only thing I can get a diagnosis for. Being a Black AFAB person who is an adult, my chances of even being able to get in for autism testing are slim to none.”

#### Theme 3. Consequences of multiple discrimination in health care settings

Participants described the negative impact of anticipating and experiencing multiple, intersecting forms of interpersonal discrimination in health care settings on their health care utilization, the quality of care they received, and their mental and physical health.

##### Delaying and avoiding care

Many participants described delaying or avoiding seeking health care in response to prior and fears of future experiences of multiple and intersectional discrimination in health care settings. For example, referring to the negative impact of weight-based discrimination on seeking care, a Black gender non-conforming participant stated: “And it got to the point where I literally, for a span of about 8 months, I literally just refused to go to the doctor for anything. Because I couldn’t handle the stress and disappointment. I would cry if someone told me to go to the doctor because I couldn’t handle it. Because the sheer amount of fatphobia that I would face from doctors, would just be too much.” Further, with regard to the negative effects of experiencing cissexism in health care settings, a Latinx/e and Native transmasculine participant mentioned: “Last year I got in a car accident, and I had to go through the ER alone and it was one, super scary because I was in a car accident, and I broke my leg. But two, it was super invalidating and felt really hard because everyone was misgendering me and talking about my body in ways that didn’t feel good. I was wearing a chest binder and they wanted to cut it off of me. And I was like, ‘No, I can’t, I won’t. I can’t afford to replace it, so I will pull it off myself.’ And luckily, one nurse had a trans son, and she intervened and was like, ‘No, let them take it off.’ But just, stuff like that makes me really hesitant to go to the doctor if I don’t absolutely need to.”

Similarly, a Black gender non-conforming participant described discontinuing their care in response to a provider’s refusal to respect their gender identity: “I just stopped going to see her because she was a cis[gender] white woman, and she didn’t really get the whole trans thing. Even when I went to see her while I was on my hormones, she always misgendered me, she always used the wrong name. I had told her what the correct name was, and she didn’t listen to me.” Moreover, a Black, Latinx/e, and white non-binary participant stated: “If it doesn’t kill me then why put myself through having to correct someone on my body and my gender? Because if they’re like, ‘You’ve got to get your teeth cleaned’, I’m just going to get my teeth cleaned and you’re going to call me a man the whole time and make me uncomfortable. So why put myself through that just for a cleaning?”.

##### Erasure of social identities and lived experiences

Several participants described how experiencing multiple, intersecting forms of discrimination in health care settings led to the erasure of aspects of their social identity and lived experience—which in turn undermined the quality of care they received, including patient-provider communication and the recommendation and delivery of relevant and needed health services. For example, referring to how they negotiate their gender identity in the context of cisnormative health care settings, a Black, white, and multiracial non-binary participant explained: “I’ve been nervous lately. Like when my PCP [primary care provider] asked for my pronouns, I almost lied. Because I was like I don’t know how this is going to affect the care that I get. I don’t know, I just feel weird because I think that I feel concerned that if I tell people I’m non-binary, they’re going to think that’s weird and then think that I’m dumb and not want to…so, I’ve hidden it a lot.” Similarly, with regard to how health care providers interact with their multiple, intersecting social identities and lived experiences, a Black, white, and multiracial demi-girl noted: “It’s just like people pick the pieces that they can find comfort in. For example, like my privilege of being light skinned, they try to find comfort in that. But the things that seem exotic to them and that they don’t want to educate themselves on or that they don’t understand, they try to keep away from it as much as they can—even if that means risking my health care or risking my needs and not acknowledging them.”

##### Negative mental and physical health effects

Participants reported that accessing health care as a transmasculine young adult of color presented mental health risks that often outweighed the benefits of receiving care. For example, a Black gender non-conforming participant mentioned: “I wanted to perish, frankly. I wanted to, you know, take a nap and not wake up. It wasn’t great. It made my mental health not feel good. And it was just really frustrating. Because that – on top of all the other things that were going on with me – that she didn’t really listen to me about [this] was the straw that broke the camel’s back.” Further, referring to the negative mental health impact of experiencing cissexism in the especially vulnerable setting of gynecological care, an Asian non-binary participant noted: “She would misgender me every time I went in for surgery stuff or like check-up stuff. And I never felt comfortable correcting her because I was always half naked with my foot in the stirrups. And I felt super vulnerable because I’m also a survivor, so I had a lot of trauma around there.”

Moreover, describing the deleterious mental health effects of having to educate their health care providers about transgender health, an Asian non-binary participant stated: “The overwhelming feeling is exhaustion. So the onus is on trans people to educate their providers a lot of the time, and not the other way around. And it shouldn’t be that way.” A few participants also described physical risks associated with stigmatizing and discriminatory health care experiences. For example, referring to the negative effects of cissexism on their reproductive health, a Black, Latinx/e, and white non-binary participant stated: “A lot of things slipped by because I didn’t have the confidence or the knowledge or couldn’t get past the anxiety to explain to them that’s not what I wanted, this is not how I identify… I’m anemic, so I was losing blood a lot with this IUD [intrauterine device]. At one point I passed out and I had to go to [a clinic] to get it emergency removed.”

#### Theme 4. Strategies for challenging and resisting multiple discrimination in health care settings

Participants discussed using various strategies to challenge and resist multiple, intersecting forms of discrimination and their negative effects in health care settings.

##### Setting boundaries with health care providers

Some participants described setting clear boundaries to protect their mental health and well-being in the context of patient-provider interactions. For example, with regard to preempting any negative impact of potential weight-based discrimination, a Black, Native, and multiracial agender participant explained: “I appreciated that she wouldn’t tell me my weight. I would be like, ‘I don't want to know my weight. I don't have any intention of exercising or doing anything to modify my body weight because I’m pretty happy with how I am.’ [She was] like, ‘Okay, I can respect that. But we will keep an [eye] on things if they become concerning.’ And I was like, ‘That’s fair.’” Further, a Black, Latinx/e, and white non-binary participant mentioned: “I’d gone to this physical therapy place. [The health care providers] were like, ‘We have to do this, we have to do that.’ I was like, ‘Oh, I’m actually not comfortable doing this, because I don’t want you touching me there.’ And they were like, ‘Yeah, but all the guys do it,’ and I was like ‘But I’m not a guy,’ and she was like, ‘What do you mean?’ I had to briefly explain that I was trans and that I wasn’t comfortable doing that because my chest is not the same as cis males.”

##### Seeking care from competent health care providers with shared marginalized social positions

Most participants mentioned seeking out health care providers who were knowledgeable about transgender health and shared one or more of their marginalized social positions and related lived experiences in order to avoid discrimination and promote their comfort and well-being during clinical encounters. For example, with regard to avoiding experiencing cissexism while obtaining mental health care by seeking care from a non-binary provider, a Black agender participant noted: “I got lucky with my therapist that I see. What made me comfortable with this therapist was that we had a shared experience, we’re both non-binary people. And they actually were one of the first people to affirm that it was okay if I wanted top surgery [in a] different [way] than a lot of people, because eventually, I do want to get top surgery. But I want a reduction; I don’t want to say goodbye completely to everything. But also knowing that my therapist was sharing their experiences as well made me feel comfortable.”

Further, referring to avoiding experiencing racism and xenophobia in health care settings by receiving care from a provider of color, a Black, Latinx/e, and white non-binary participant mentioned: “I specifically wanted a doctor of color because I had a really bad experience with a white doctor. I specifically wanted to go to a place that was more diverse because there’s been two times where I’ve been asked for my Green Card. So, I specifically wanted a doctor of color, which I did get. I’m very lucky. I love her so much.” In contrast, a few participants mentioned being concerned about receiving care from a health care provider of color due to fears that they might not be affirming or accepting of their gender identity. For example, an Asian non-binary participant observed: “I think there’s this perception that people who are white American are more LGBTQ-friendly, and whoever you are, you kind of internalize that. And so, I think if a doctor is someone who isn’t white American, I’m like, ‘Are you going to be queer-friendly?’ And it’s probably not fair. That’s a stereotype, just totally a stereotype and a bias I have.”

##### Engaging in self-advocacy

Many transmasculine young adults of color in our study mentioned having to advocate for themselves in order to receive competent care, including by asking questions about their care, requiring that their health care experiences be documented, and requesting to change health care providers. For example, with regard to asking questions about their care, a Black non-binary participant explained: “I am someone where I really take my health care seriously. And so when I don't understand something, I would like to ask a doctor. Like, ‘Oh, we’re going to test you for this,’ and if I don’t know what that means, I’m like, ‘Okay, what is that?’ And they tell me.” Moreover, referring to ensuring that their health care experiences and care decisions are documented, a Black and Latinx/e non-binary participant noted: “I walk into every room and ask for a scribe to be there because I do not trust my health care providers. I don’t talk to any health care provider without someone in the room who is transcribing exactly what is happening, who will answer me when I ask them if they noted something in my chart, who will read things back to me before I leave.”

Similarly, a Black gender non-conforming participant explained: “Before I knew that I could [ask], ‘Oh, can you please note on my chart that you refused to give me treatment for my nausea that’s seriously affecting my health?’ Before I knew that was an option, I just wouldn’t go [to the doctor]. Now that I know that there’s a way to keep medical professionals that I see accountable, I might go to the doctor. And I feel like I finally have a tool to really help me get the care that I need.” Lastly, referring to requesting a different health care provider after experiencing cissexism, a Black, Latinx/e, and white non-binary participant mentioned: “This is the person who tried to change my name in the system back to my deadname, and I was like, ‘Have you ever heard of a trans person?’ And she was like, ‘You’re trans?’ And I was like, ‘Have you ever heard of a trans person?’ And she was like, ‘Not’til you, I guess.’ And I was like, ‘Who in your office has?’ She said, ‘I guess so-and-so.’ I was like, ‘You need to transfer me to them.’ So I did get transferred to this person. She changed my name back to what it’s supposed to be and helped me get back on my insurance.”

##### Drawing on peer support during health care visits

Some participants mentioned having partners or friends accompany them to health care visits to provide emotional support and advocate on their behalf. For example, a Black, Latinx/e, and white non-binary participant noted: “So I’m really scared of the doctors – most providers, so I usually have someone come with me. And if they [partner] see that I am not going to bring it up, because I’m too nervous, they will bring it up. So, my partner has been really good about that because usually we’ll start talking and all of a sudden, they [provider] will slip a ‘he’ pronoun and my partner is like, ‘Actually it’s ‘they’.’ And that will initiate the conversation of me being more comfortable to tell them that I’m non-binary.” Moreover, a Black and Latinx/e non-binary participant stated: “Having someone in the room while my doctor tried to force an IUD [intrauterine device] on me, that was great for me because it just made me realize that I didn’t dissociate and hallucinate the whole experience.” Lastly, a few participants expressed that, when they were unable to find someone to accompany them to a health care visit, they delayed or avoided care. For example, a Black, Latinx/e, and white non-binary participant mentioned: “That’s why I cancelled my last two dentist appointments. Because my partner couldn’t go, and I was too scared to [go alone].”

##### Obtaining health information through social networks

Many participants mentioned that, in the context of stigmatizing and discriminatory health care experiences, they instead turned to other transgender and gender diverse people for health information that meets their needs by accessing either in-person or online social networks. For example, a Black gender non-conforming participant explained: “That’s probably the reason why I don’t go for preventative care stuff. Because various aspects of my identity form a barrier to receiving a specific type of care that I would need. So, I just don’t talk about it, and I just don’t go. I know enough people that have similar experiences that we all just kind of form a little community network of like, ‘Okay, I’m sick, what do I do?’ ‘Oh, I do this.’ ‘Okay, I’m going to try that.’ And then it works.” Further, an Asian non-binary participant noted: “I feel like the queer community at large, at least the ones that I’ve been a part of, are very vocal about the importance of [STI testing]. And that’s primarily where my education’s come from, from the community, not from a doctor, not from online, regarding STIs.” They continued: “I mean, for a lot of queer and trans people, it’s like, if we don’t protect each other, who will?”

##### Recommending critical health care provider training

Several participants recommended that, in order to address how multiple, intersecting forms of discrimination simultaneously undermine the health care experiences of transmasculine people of color, health care providers receive critical training in recognizing and addressing cissexism, racism, and other forms of discrimination in health care settings. For example, an Arab and multiracial transmasculine participant suggested: “It’s critical teaching physicians about the racialized history of STI stigma and research. There’s the Tuskegee trials and all these [awful things] that doctors don’t know about. And so, trying to mitigate that by actually giving explicit and race critical and gender critical education to physicians.” Moreover, a Black, white, and multiracial demi-girl recommended: “For the health care providers, I think it’s important that they get effective training and regular training, not just like, ‘Oh I took a 30-min little class that a cisgender straight person taught me about cultural competence with LGBT people,’ and also the teacher was white and talked a little bit about racism but that they didn’t experience [it]. I just hope that health care providers can get more effective training and direct knowledge from people that actually have experiences of it and include our voices.”

## Discussion

Using thematic analysis, we found that transmasculine people of color experience pronounced barriers to accessing physical and mental health care, all of which can be linked to structural cissexism, structural racism, and capitalism. Specifically, the participants in our study reported having trouble identifying health care providers, including obstetrician/gynecologists, with expertise in transgender health, which is linked to pervasive structural and institutional cissexism in society in general and the health care system in particular [50–54]; challenges obtaining care from providers who shared their gender identity, race/ethnicity, and/or other marginalized social positions, which is linked to structural and institutional cissexism, racism, and other forms of discrimination [55, 56]; and difficulty meeting the high financial costs of health services, which is linked to the market-based nature of health care in the U.S [57–59]. Additionally, study participants reported anticipating and experiencing multiple, intersecting forms of interpersonal discrimination—including cissexism, racism, weight-based discrimination, and ableism—in various health care settings. While most participants discussed these fears and experiences of discrimination separately, many also described how they simultaneously impacted their health care experiences in compounding ways, in line with intersectionality [10–14]. Our findings align with and build on those of Howard et al., who found that transmasculine people of color experienced discrimination in relation to both gender identity and race/ethnicity in health care settings [19]. Similarly, the authors also found that, although participants tended to describe these two forms of discrimination separately and mentioned that their dominance varied across clinical encounters, the health care experiences of transmasculine people of color were simultaneously and uniquely impacted by both cissexism and racism, among other forms of discrimination, as shown in our study [19].

Of note, our study elucidates the unique and specific impact of anti-Black racism on the health care experiences of Black transmasculine people in particular, as well as the mutually constitutive and compounding ways in which anti-Black racism intersects with cissexism and weight-based discrimination in the health care system. Specifically, we found that all Black participants experienced racism during clinical encounters, which underscores the persistent and pervasive nature of anti-Blackness in U.S. society in general and in the health care system in particular. Indeed, critical race theorists explain that racism, especially anti-Black racism, underpins all U.S. systems and institutions, including but not limited to the education, employment, housing, criminal legal, and health care systems [60, 61]. Moreover, Washington, Roberts, and others have demonstrated how U.S. medicine, which espoused and propagated eugenics, was developed through the exploitation of and experimentation on Black individuals, from slavery to the present [62–64]. Lastly, scholars have also shown the mutually constitutive nature of anti-Black racism and other forms of oppression, including transphobia, anti-fatness, and ableism, among others, which subjugate and disproportionately harm those who experience them simultaneously [65, 66]. Thus, our study provides an empirical illustration of how these historical and contemporary processes undermine the health care—and in turn, the health—of Black transmasculine people in particular, whose experiences in the health care system are shaped by intersections of anti-Black racism, cissexism, weight-based discrimination, and ableism.

Similarly to other quantitative [8, 67] and qualitative [19, 68] research studies, we also found that experiences of discrimination in health care settings were linked to the denial of, delays in, or avoidance of care and the receipt of low-quality care among transmasculine people of color. Further, in line with other research conducted among predominately cisgender populations, we also identified negative effects of health care discrimination on participants’ mental and physical health [9, 69, 70]. Lastly, in line with other studies conducted among predominately white transmasculine individuals [50, 71], our findings indicate that transmasculine people of color actively use a range of strategies to challenge and resist the multiple forms of discrimination they face in health care settings. Specifically, study participants reported establishing boundaries with health care providers, seeking care from providers with expertise in transgender health and who share at least one of their social marginalized positions, self-advocacy during clinical encounters (including asking questions and requiring documentation of the visit), ensuring peer support during health care visits, and obtaining health information through social networks of transgender and gender diverse peers [71].Additionally, participants recommended that health care providers receive critical, historically-informed training in recognizing and addressing cissexism, racism, and other forms of discrimination in health care settings.

Our findings should be interpreted in the context of several limitations. First, our sample primarily consisted of individuals who had received at least some college education and were enrolled in a private health plan; thus, findings may not reflect the experiences of transgender and non-binary AFAB people with less than a college-level education or those enrolled in a public health plan or lacking health insurance. Second, our study was limited to young adults aged 18–25 years; as such, our findings may not be applicable to younger or older transmasculine people of color. Third, focus groups took place in the greater Boston area, thus findings may be less applicable or transferable to those who live in geographic areas with other social (e.g., more conservative gender norms and sexual attitudes), economic (e.g., lower levels of educational attainment), political (e.g., no legal prohibition of gender identity-related discrimination), and health policy (e.g., located in a state with no Medicaid expansion) climates. Fourth, focus groups were not stratified by race/ethnicity or gender identity, so findings may have masked differences among racial/ethnic and gender identity subgroups of transmasculine young adults of color. Fifth, our findings pertain to experiences of interpersonal discrimination in health care settings and do not examine the influence of institutional discrimination in the health care system or of structural discrimination in society more broadly, which shapes individuals’ health care experiences as well as the nature, occurrence, and impact of interpersonal health care discrimination. Lastly, we were not able to examine transmasculine people of color’s experiences of heterosexism, xenophobia, and other forms of discrimination in our study. Thus, future quantitative, qualitative, and mixed-methods research studies that investigate how various multiple and intersectional forms of discrimination at multiple levels and in multiple settings differentially and simultaneously affect the health care experiences of specific subgroups of transmasculine people of color from diverse gender identity and racial/ethnic as well as socioeconomic, age, geographic sexual orientation, and nativity backgrounds, among others, are needed.

Our study on the experiences of multiple, intersecting forms of discrimination in health care settings has important implications for practice and policy. First, as underscored by many participants in our study, there is a pressing need for health care facilities to hire, promote, and retain transgender and gender diverse health care providers and health care providers of color, including providers with both marginalized gender identity and racial/ethnic positions, at all levels of the institution. Indeed, research shows that receiving care from a provider who shares one or more marginalized social positions with multiply marginalized patients can promote patient comfort during clinical encounters, as well as the delivery of high-quality care [21, 72–75]. However, health care institutions should ensure that marginalized health care providers and leaders are supported in their work and that robust mechanisms are in place to identify and meaningfully address bias, prejudice, and discrimination directed toward them when they occur. Second, some participants called for health care providers to receive critical, historically-grounded training on the impact of cissexism, racism, and other intersecting forms of discrimination on health care. It is possible that these trainings may decrease the burden transmasculine people of color face in having to educate providers about their gender-specific health concerns and health care needs [2]. However, research shows that provider training has limited and mixed results on improving provider knowledge and delivery of clinical services to marginalized populations, including transgender and gender diverse individuals [76]. For example, in a 2019 study, Stroumsa et al. found that providers’ knowledge of transgender health was associated with transphobia but not with formal or informal education on the topic [77].

Thus, efforts are needed to address cissexism, racism, weight-based discrimination, ableism, and other intersecting forms of discrimination in clinical encounters, health care institutions and systems, and society in general. At the interpersonal level, research suggests that using a shared-decision making approach rooted in the provision of person-centered [78], structurally competent [79], and culturally humble [80] care may be beneficial to improving the health care experiences and promoting the health of transmasculine individuals of color [81]. Such an approach would ensure that providers listen to and center transmasculine patients of color’s health and health care concerns, needs, and experiences at the intersection of multiple dimensions of discrimination and in the context of other societal factors that shape their health and lives, including housing, employment, and food security [81]. Moreover, this approach would also involve providers avoiding making assumptions about transmasculine people of color’s behaviors, concerns, and needs, using accurate language to refer to patients and their bodies, ensuring that only information that participants would like to document is included in the visit notes, and that supportive individuals of patients’ choice are allowed to attend clinical visits [50, 71, 81–83].

At the institutional level, efforts are needed to dismantle practices, rules, regulations, systems, and norms that perpetuate and reinforce cissexism, racism, weight-based discrimination, ableism, and other forms of discrimination and implement systems that promote equity and respect towards transmasculine individuals of color and other multiply marginalized populations [84]. Of note, research suggests that applying a shared decision-making framework that centers the lived experiences and health care experiences of transmasculine people of color to the organizational context of health care institutions, including their workflows, health information technology, organizational structure and culture, resources and clinic environment, training and education, and incentives and disincentives, may help improve health care experiences and health outcomes in this and other marginalized populations [84]. Further, health care institutions can create searchable directories of health care providers with lived experiences of and competence in providing care at the intersections of gender identity and expression, race/ethnicity, and other dimensions of social position and ensuring the inclusion of transmasculine people of color and other marginalized groups in health education and other materials [73]. Moreover, health care institutions can partner with community-based organizations that serve transmasculine individuals of color to deliver health services in trusted community-based settings, which may facilitate access to and utilization of high-quality health care in these communities [21, 71].

Even still, addressing multiple, intersecting forms of discrimination in health care settings will require the creation of new health care institutions and community-based organizations that center the lived experiences of transmasculine people of color from the beginning and provide tailored, person-centered, and structurally competent care that challenges structural and interpersonal cissexism, racism, and other forms of discrimination as well as capitalism in health care [85]. Lastly, ensuring access to high-quality health care among transmasculine people of color will necessitate repealing discriminatory laws, policies, and regulations and challenging social norms that promote and reinforce cissexism, racism, weight-based discrimination, ableism, and other social inequities at all levels of society [86–88]. Further, efforts to establish and promote structures, systems, practices, and norms that foster equity and social justice at the intersection of multiple dimensions of social inequality in all sectors of society through advocacy, community organizing, and participatory social movements that center the most marginalized are urgently needed to ensure access to high-quality care and foster health and well-being among transmasculine people of color and other multiply marginalized populations [86, 88].

## Supplementary Information

**Additional file 1.** Transmasculine People of Color Sexual Health Care Study: Focus Group Discussion Guide.

## Data Availability

The data used in the present study are not available per our IRB research protocol. For additional information, please contact Dr. Madina Agénor at madina_agenor@brown.edu.
