# Supplementary material for: Experiences of and resistance to multiple discrimination in health care settings among transmasculine people of color
Source: BMC Health Serv Res. 2022 Mar 21;22:369. doi: 10.1186/s12913-022-07729-5 (PMC8935683; doi:10.1186/s12913-022-07729-5)
Supplement: Supplementary file 1 — Additional file 1. Transmasculine People of Color Sexual Health Care Study: Focus Group Discussion Guide. [file 12913_2022_7729_MOESM1_ESM.docx]

**Transmasculine People of Color Sexual Health Care Study:**

**Focus Group Discussion Guide**

**Welcome**

1. Welcome participants to the focus group room and ask them to sign in and complete a name tent (pseudonym of their choice and pronouns).
2. Offer participants food and drinks and let them know where bathroom is.
3. Introduce moderator and notetaker to the participants with name and pronouns.
4. Ask participants to introduce themselves using chosen pseudonym and pronouns and share why they’re here today.
5. Draw seating chart and assign each participant a study ID number (notetaker)
6. Take notes identifying each participant with first few words of sentence and study ID number (notetaker)

**Informed consent process**

1. Review informed consent form with participants (paraphrase in own words, making sure to hit all key points).
2. Make clear that what is said in this room should stay in this room and that participants should share as much or as little as they feel comfortable doing in a group setting.
3. Ask participants if they have any questions and answer all questions; assistant moderator may answer questions privately if requested.
4. Ask participants to sign and date both copies of the informed consent form once all questions have been answered.
5. Collect both signed and dated informed consent forms from participants.
6. Sign and date both informed consent forms.
7. Keep one form for each participant (store in large envelope) and return one form to each participant.
8. Informed consent forms should have participant study ID number (same as the one on the seating chart) at top of each page.

**Focus Group Guide Questions**

1. **Access to Health Information and Health Care**
2. Since identifying as transmasculine, what do you do when you need **information** about a health issue?
   1. Probe for what health issues has looked for information on and why.
   2. Probe for sources of health information, including peers, partners, organizations, parents, providers, and Internet, has turned to and why.
      1. Probe for what sources of information are preferred and why.
   3. Probe for satisfaction with health information, including but not limited to in relation to transgender status.
3. Since identifying as transmasculine, where do you go when you need **health care**?
   1. Probe for what issues usually seek health care for (e.g., transition related care, preventive care).
   2. Probe for health care setting (e.g., community health center, private doctor’s office) and provider type (e.g., primary care provider, specialist) usually seek care from.
   3. Probe for how decide whether, where, and from whom to get health care, including but not limited to in relation to transgender status.
   4. Probe for any challenges encountered in identifying and accessing health care, including with health insurance and being denied health care by a provider.
   5. *If doesn’t go anywhere, ask:* What are some reasons why you haven’t gone anywhere when you need or want health care? What do you do instead?
4. **Health Care Experiences**
5. Tell me about the last time you obtained **health care** for yourself since identifying as transmasculine.
   1. Probe for type of health care sought (e.g., transition-related care, preventive care, reproductive health care).
   2. Probe for type of health care setting and health care provider.
   3. Probe for how and why chose that setting and provider, including but not limited to in relation to gender identity and transgender status.
   4. Probe for experience communicating and interacting with health care providers and staff.
   5. Probe for whether and how involved in making decisions about own health care.
6. Tell me about a time when you **disclosed** your transmasculine status to a health care provider since identifying as transmasculine.
   1. Probe for how the topic has come up, what was said, provider reactions, and participant feelings during and after.
   2. What, if anything, would you change about this experience?
   3. Probe for whether this particular experience was similar or different from other experiences.
   4. *If a participant has not disclosed their transgender status to a provider, ask*: What are some reasons why you haven’t disclosed your transgender status to a health care provider?
7. Tell me about a time when your **transmasculine status** affected your health care.
   1. Probe for influence of transgender status on experience in waiting room and interactions with office staff and health care providers, including patient-provider communication and shared decision-making.
   2. Probe for experiences of differential treatment.
8. Tell me about a time when your **race/ethnicity** affected your health care.
   1. Probe for influence of race/ethnicity on experience in waiting room and interactions with office staff and health care providers, including patient-provider communication and shared decision-making.
   2. Probe for experiences of differential treatment.
9. Tell me about a time when **other aspects** of your identity affected your health care.
   1. Probe for age, sexual orientation, gender expression, health insurance status, education, immigrant status, disability status, and language.
   2. Probe for influence on experience in waiting room and interactions with office staff and health care providers, including patient-provider communication and shared decision-making.
   3. Probe for experiences of differential treatment.
10. How much do you **trust** health care providers to help you with your health concerns?
    1. Probe for reasons for mistrust.
11. **HIV/STI Perceptions and Communication**
12. When you hear ***HIV***, what comes to mind?
    1. Probe for beliefs, thoughts, feelings, and comfort.
13. When you hear ***sexually transmitted infection*** or ***STI***, what comes to mind?
    1. Probe for beliefs, thoughts, feelings, and comfort.
14. Since identifying as transmasculine, what do you do when you need **information** on HIV and STIs?
    1. Probe for both HIV and STIs (e.g., Chlamydia, gonorrhea, herpes, HPV).
    2. Probe for specific topics has looked for information on (e.g., HIV/STI risk, prevention, testing, treatment) and why.
    3. Probe for sources of information, including peers, partners, organizations, parents, providers, and Internet, has turned to and why.
       1. Probe for what sources of information are preferred and why.
    4. Probe for satisfaction with information, including but not limited to in relation to transgender status.
    5. *If participant hasn’t looked for information on HIV or STIs*, ask: What are some reasons why you haven’t looked for information about HIV or STIs?
15. Tell me about your experience **discussing** HIV and STIs with health care providers since identifying as transmasculine.
    1. Probe for both HIV and STIs (e.g., Chlamydia, gonorrhea, herpes, HPV).
    2. Probe for specific topics discussed with health care providers (e.g., HIV/STI risk, prevention, testing, treatment).
    3. Probe for types of settings (e.g., community health center, private doctor’s office) where and providers (e.g., primary care provider, specialist) with whom discussed HIV and/or STIs.
    4. Probe for feelings associated with discussing HIV and/or STIs with health care providers (e.g., fear, shame, anxiety).
    5. What have you liked the *most* about your experience discussing HIV and/or STIs with health care providers since identifying as transmasculine?
    6. What have you liked the *least* about your experience discussing HIV and/or STIs with health care providers since identifying as transmasculine?
    7. Probe for particularly good and particularly bad experience discussing HIV and/or STIs with a health care provider since identifying as transmasculine.
    8. *If participant hasn’t discussed HIV or STIs with a provider*, ask: What are some reasons why you haven’t talked about HIV or STIs with a health care provider? Is this a topic you would like to discuss with a provider? Why or why not?
    9. Who else have you spoken with about HIV and STIs? Probe for parents, siblings, peers, and partners.
16. **HIV/STI Testing Perceptions and Experiences**
17. Since identifying as transmasculine, what have you heard from others about whether or not transmasculine individuals need to get **tested** for HIV and STIs?
    1. Probe for both HIV and STIs.
    2. Where/from whom did you hear this?
18. Since identifying as transmasculine, do you personally think transmasculine individuals need to get **tested** for HIV and STIs? Why or why not?
    1. Probe for both HIV and STIs.
    2. Probe for reasons why transmasculine people need or don’t need to get tested.
    3. Probe for whether think transmasculine people are at risk of HIV and STIs.
19. What are some reasons why transmasculine individuals **may not get tested** for HIV and STIs?
    1. Probe for both HIV and STIs.
    2. Probe for barriers related to stigma, discrimination, insurance, gender identity, racism, lack of competent providers, power dynamics in relationships, cost.
20. Tell me about your experience getting **tested** for HIV and STIs since identifying as transmasculine.
    1. Probe for both HIV and STIs (Chlamydia, gonorrhea, herpes, HPV).
    2. Probe for types of settings where (e.g., community health center, private doctor’s office) and providers (e.g., primary care provider, specialist) from whom received an HIV and/or STI test.
    3. Probe for whether self-swabbing in office or at home was an option and if not, if would have liked this option.
    4. Probe for how decide whether, where, and from whom to get HIV and/or STI tests.
    5. Probe for feelings associated with obtaining HIV and/or STIs tests (e.g., fear, shame, anxiety).
    6. Probe for satisfaction with services, including interactions with office staff and health care providers.
    7. What have you liked the *most* about your experience obtaining HIV and/or STIs tests since identifying as transmasculine.
    8. What have you liked the *least* about your experience obtaining HIV and/or STIs tests since identifying as transmasculine.
    9. Probe for any challenges identifying, accessing (including with health insurance vis-à-vis gender markers and being denied service by a health care provider), and/or utilizing (including differential treatment based on gender identity, race/ethnicity, or another social identity) HIV/STI testing services.
    10. Probe for particularly good and particularly bad experience obtaining HIV and/or STIs tests since identifying as transmasculine.
    11. *If participant hasn’t been tested for HIV or STIs*, ask: What are some reasons why you haven’t been tested for HIV and/or STIs? Are you interested in obtaining an HIV and/or STI test? Why or why not?
21. **Recommendations**
22. What type of **information** would you like to receive on HIV and/or STIs?
    1. Probe for type of information would like related to both HIV and STIs (e.g., Chlamydia, gonorrhea, herpes, HPV), including specific topics of interest (e.g., risk, testing, treatment).
    2. In what format would you like to receive this information?
       1. Probe for training, pamphlet, website, app.
    3. From whom would you like to receive this information?
       1. Probe for providers, community organizations, parents, peers.
    4. At what age(s) would you like to receive this information?
23. What should **health care facilities** do to ensure transmasculine individuals receive high-quality HIV and/or STI testing services?
    1. Probe about clinic environment, office staff, health care providers (including communication, training, and knowledge), intake forms, insurance coverage, physical exams.
    2. Probe about availability, access, and utilization of services.

**Wrap-up**

*That’s it for the specific questions that I have for you. Is there anything else you would like to share about your views and experiences related to health care in general or HIV and STI testing in particular? Is there anything we didn’t discuss today that you think we should have?*

*Thank you very much for participating in this focus group. We really appreciate your time. Before you go, we have a brief, anonymous survey for you to fill out so that we can learn more about your background and experiences.*
